# Persistence or Clearance of Human Papillomavirus Infections in Women in Ouro Preto, Brazil

**DOI:** 10.1155/2013/578276

**Published:** 2013-11-05

**Authors:** P. M. Miranda, N. N. T. Silva, B. C. V. Pitol, I. D. C. G. Silva, J. L. Lima-Filho, R. F. Carvalho, R. C. Stocco, W. Beçak, A. A. Lima

**Affiliations:** ^1^Laboratório de Genética, Instituto Butantan, Avenida Vital Brasil 1500, 05503-900 São Paulo, SP, Brazil; ^2^Departamento de Análises Clínicas Escola de Farmácia, Universidade de Ouro Preto (UFOP), Rua Costa Sena 171, 35400-000 Ouro Preto, MG, Brazil; ^3^Laboratório de Tocoginecologia, Departamento de Ginecologia, Universidade Federal de São Paulo, 04039-032 São Paulo, SP, Brazil; ^4^Laboratório de Imunopatologia Keizo Asami (LIKA), Universidade Federal de Pernambuco (UFPE), 50670-901 Recife, PE, Brazil; ^5^Programa de Pós-Graduação Interunidades em Biotecnologia, Universidade de São Paulo, 05508-900 São Paulo, SP, Brazil; ^6^Departamento de Biologia, Universidade Federal da Integração Latino-Americana (UNILA), 85867-970 Foz do Iguaçú, PR, Brazil

## Abstract

Persistent high-risk (HR) human papillomavirus (HPV) infection is necessary for development of precursor lesions and cervical cancer. We investigate persistence and clearance of HPV infections and cofactors in unvaccinated women. Cervical samples of 569 women (18–75 years), received for routine evaluation in the Health Department of Ouro Preto, Brazil, were collected and subjected to PCR (MY09/11 or GP5+/6+ primers), followed by RFLP or sequencing. All women were interviewed to collect sociodemographic and behavioral information. Viral infection persistence or clearance was reevaluated after 24 months and was observed in 59.6% and 40.4% of women, respectively. HPVs 16, 33, 59, 66, 69, and 83 (HR) were the most persistent types whereas HPVs 31, 45, and 58 were less persistent. Clearance or persistence did not differ between groups infected by HPVs 18, 53, and 67. In low-risk (LR) types, HPV 6 infected samples were associated with clearance, while HPV 11, 61, 72, or 81 infected samples were persistent in the follow-up. No statistically significant association was detected between persistent HPV infections and sociodemographic and behavioral characteristics analyzed. To study persistence or clearance in HPV infection allows the identification of risk groups, cofactors, and strategies for prevention of cervical cancer.

## 1. Introduction 

 Human papillomavirus (HPV) infections are the most commonly diagnosed sexually transmitted disease. More than 100 HPV types have been identified. They infect the skin squamous epithelia and mucosa and usually cause benign papillomas or warts. Persistent infection with oncogenic high-risk HPV causes all cervical cancers, most anal cancers, and a subset of vulvar, vaginal, penile, and oropharyngeal cancers [1–3].

The major steps in cervical carcinogenesis include HPV infection, HPV persistence for a certain period of time, progression to precancer, and invasion [4, 5].

HPV infection alone may not be sufficient to cause cervical cancer, and other factors influence the risk of progression to cervical cancer. High parity, smoking, long-term use of oral contraceptives, sexual behavior, genetic factors, and coinfection with other sexually transmitted infectious agents such as herpes simplex virus 2 (HSV-2) and *Chlamydia trachomatis *have been established as cofactors for cervical cancer among women with persistent infections [6–9].

Although these environmental and genetic factors are considered to act as HPV cofactors contributing to progression from viral infection to cervical cancer, few prospective studies have addressed the roles that these risk factors play in the natural history of precursor lesions. The knowledge of epidemiology and natural history of HPV infection (acquisition, clearance, and persistence) is relevant and can direct interventions to prevent preneoplastic lesions and cervical cancer [4].

There is evidence that most HPV infections are transient and clear spontaneously within 12–24 months after first detection. Persistent HPV infection with elevated risk of cervical cancer occurs in only a small percentage of virus infected women [5, 10]. The reasons of this fact are still unknown.

The importance of HPV clearance/persistence has been recognized recently, and the number of studies addressing these issues has increased substantially during the past few years. However, data are still incomplete and in part inconsistent as to the cofactors that regulate these events [11, 12].

In Ouro Preto, MG, Brazil, a recent study showed prevalence of HPV infections in women with normal cervical cytology. Different subtypes of high-risk HPV (mainly HPV 16) were predominant. Furthermore, age, literacy, residence, marital status, smoking status, and number of sexual partners were independently associated with HPV infection [13].

HPV-positive women in this prevalence study have been accompanied to assess the natural history of viral infection. Therefore, the aim of this study was to investigate persistence and clearance of HPV infections in unvaccinated women and cofactors correlated with such events. 

## 2. Material and Methods

*Ethical Statements. *The protocols used in this study were approved by the Ethical Committee in Research of the Universidade Federal de São Paulo (number 0832.08) assigned by the president of this committee. 

The sample was composed of 569 women (aged 18 to 75 years) living in Ouro Preto, MG, Brazil. Women were randomly selected when they visited health units for routine gynecological evaluation and they were not enrolled in the study if they were pregnant, diagnosed with mental incompetence, or diagnosed with carcinoma, except the cervical cancer. This project was reviewed and approved by the Institutional Ethic Committee, and informed consent was obtained from private interviews at the time of gynecological evaluation.

Sociodemographic characteristics, smoking status, alcohol consumption, bracken fern diet, and sexual and reproductive behavior were obtained from private interviews with the women at the time of the gynecological evaluation. Subjects were considered as nonsmokers or nonalcohol consumers if they have never smoked or drunk. Speculum examination was performed by gynecologists; cervical samples were obtained using an extended tip “Ayre” spatula. The conventional cervical smear was spread onto a glass slide and the tip of the spatula was broken off and placed in a container containing preservation solution for HPV testing.

### 2.1. HPV-DNA Typing

For the extraction of DNA from cervical samples, we used a GenomicPrep Blood kit (GE Healthcare), with proteinase K digestion. HPV was detected by the amplification of DNA by using a standard polymerase chain reaction (PCR) protocol with L1 consensus primer pair MY09 and MY11, which promotes amplification of an approximately 450 bp product and can detect more than 40 distinct low- and high-risk genital HPV types. Positive and negative controls were used in all tests. The amplification was performed according to the following protocol: 94°C for 30 s, 51.5°C for 30 s, and 72°C for 30 s for 35 cycles, followed by a final step at 72°C for 7 min [14, 15]. The *β*-actin gene primers were used as internal controls.

We used RT-PCR with primers GP5+/6+ for HPV detection in samples with no conclusive diagnosis using primers MY09/11. The reactions were performed using SYBR Green PCR Master Mix (Applied Biosystems) and conducted in ABI 7000 Applied Biosystems.

HPV-DNA-positive specimens were typed by restriction fragment length polymorphism (RFLP), according to Bernard et al. 1994 [16]. Samples with positive HPV viral types not identified by RFLP were identified by sequencing of the PCR product using BigDye Terminator kit (Applied Biosystems). Sequences were generated on ABI model 377 automated sequencer (Applied Biosystems) and aligned to HPV sequences at Genbank using BioEdit Sequence Alignment Editor 7.0.9.0. 

### 2.2. Follow-Up Study

HPV infection was detected in 133 women, who were invited to participate in a follow-up study, consisting of a second cervical scrape specimen collection 24 months from first recruitment, using the same procedure as the initial examination. 89 women (66.9%) completed the survey and were tested for HPV in 24 months. 44 women (33.1%) were lost in the follow-up. 

After 24 months (second cervical scrape specimen for cytology and HPV testing), all women were interviewed again, with the same questionnaire used in first collection, and the information was updated. 

 Persistent type-specific HPV infection was defined as the detection of the same HR-HPV type at both examinations (first study and follow-up study). Clearance was defined as the proportion of women who were initially HR-HPV positive (first collection), but the same HR-HPV type was not found at the follow-up [12]. 

### 2.3. Statistical Analysis

Descriptive statistics were used for the analysis of persistence and clearance of type-specific HPV infection. Particularly, Pearson's *x* ^2^ test was used to verify the association between all independent variables and the response variable. *P* < 0.05 was considered statistically significant. Statistical analyses were performed using SPSS software for Windows (version 18.0).

## 3. Results and Discussion

### 3.1. Results 

#### 3.1.1. Profile of the Initial Sample

Analyses of this study were performed with samples of 89 women, mean age of 37.7 (±12.6) years.

These samples were composed predominantly of women with the following characteristics: age ≥30 years (68.5%); resident in urban areas (76.4%); only one sexual partner in lifetime (51.7%); no use of oral contraceptives (52.8%); alcohol consumption (61.8%); 1–5 pregnancies (66.3%); bracken fern consumption in usual diet (68.5%); smoking (40.4%). Only 20.2% of women in the group had early onset of sexual activity (≤15 years). 

Simple infections, by a single viral type, were predominant (80 women, 89.9%) and multiple HPV infections were detected in 10.1%.

High-risk (HR) HPV types (mainly HPV16) were more prevalent in the study samples. HR-HPV was detected in 55 (61.8%) women, indeterminate risk (IR) HPV in 17 (19.1%) and low risk (LR) in 17 (19.1%) women.

#### 3.1.2. Evaluation after 24-Month Follow-Up

Among the 89 women HPV positive at baseline, 53 (59.6%) had persistent infection and 36 (40.4%) had clearance of the infection after 24 months.

Table 1 shows clearance or persistence of viral infection in relation to number and risk of HPV types. 

 Most women with multiple infections (77.8%) showed persistence after 24 months of follow-up. However, presence of multiple infections was not statistically associated with persistent HPV infection (Table 1). 

 Similarly, in relation to risk oncogenic HPV types, no statistical difference was observed between persistence of viral infection and HR-HPV types. The infection persisted after follow-up in 61.8% of samples infected by HR-HPV and in 70.6% by LR-HPV (Table 1).

 Among specific HR-HPV types, HPV 16, 33, 59, 66, 69, and 83 were the most persistent types whereas HPVs 31, 45, and 58 were the less persistent. Clearance or persistence did not differ between groups infected by HPVs 18, 53, and 67 (Table 2). 

 Regarding specific LR-HPV types, HPV 6 infected samples were associated with clearance. In contrast, HPV 11, 61, 72, or 81 infected samples were associated with persistent infections in the 24-month follow-up (Table 3).

Table 4 shows that no statistically significant association was detected between persistent HPV infections and sociodemographic and behavioral characteristics evaluated. 

## 4. Discussion

 We have analyzed the prevalence of HPV infection in women presenting normal cervical cytology [13] and in women living in rural and urban areas of Ouro Preto, MG, Brazil [17]. HPV-positive women were followed for 24 months. In general, persistence of HPV infection was 59.6% and clearance was 40.4%. It is believed that HPV infections “clear” within 2 years in more than 90% of individuals [5, 18–20]. Therefore, the percentage of HPV clearance was low in our study but similar results were also obtained by Banura et al., 2010 [21], and Guo et al., 2010 [22]. 

 Considering only HR-HPV infections, the persistence was 61.8% and clearance was 38.2%. This result was similar to that reported in studies in Brazil [23] and other countries [12, 24, 25]. 

 However, unlike what is expected we observed high persistence (70.6%) and low clearance (29.4%) of LR-HPV infections, proportions similar to those obtained for HR-HPV types. This may be due to the small number of women with each viral type or time of follow-up. Banura et al., 2010 [21], showed clearance LR-HPV ranged between 50% and 100%. Other studies also observed that clearance proportion was similar in HR-HPV and LR-HPV [21, 26]. We found that HPV 16, 33, 59, 66, 69, and 83 infections were the most persistent. Studies have reported that HPV 16 was significantly more likely to persist than were all other viral types [21, 25, 27]. Moreover, Sammarco et al., 2013 [12], observed that HPV types with the highest likelihood of persistence were 31, 39, and 73, whilst HPV 16 was the least persistent. Persistent infections have a higher risk of progression to precursor lesions and cervical cancer.

 In this study, most of women with multiple infections showed persistence after 24 months of follow-up. Similar results were obtained by Trottier et al., 2008 [28], Nielsen et al., 2010 [27], Castle et al., 2011 [29], and Schmeink et al., 2013 [26], who reported that infection with multiple HPV types is associated with longer persistence of HPV infection. This association was not observed by Sammarco et al., 2013 [12].

 In the literature, associations of HPV persistence or clearance with cofactors vary widely due to the sample size, population characteristics, and study design. In this study, there was no significant association between HPV persistence or clearance and the analyzed cofactors. Schmeink et al., 2013 [26], suggest that HPV clearance is mainly related to the host immune response or other intrinsic host factors and not to present behavior factors.

 There are reports that women with persistent HR-HPV infections were younger than those who cleared their infections [12, 30, 31]. Others showed that persistence increased with age [29]. However, no association between HPV persistence and age was found in this study as in Trottier et al., 2008 [28], and Muoz et al., 2009 [32].

 Likewise in this work, some studies found no association between persistent HPV infection and smoking, number of sexual partners, oral contraceptive use, and high parity [12, 26, 27, 30, 33]. 

 Persistent high-risk HPV infection also was associated with high viral load [22, 34]. 

The main limitations of this study were sample size and follow-up period. This prevented extensive discussion of some results and direct comparison with other studies. Furthermore, it is important to emphasize that our data were based solely on the detection of DNA in cervical samples. 

HR-HPV persistence plays a key role in the progression of preneoplastic lesions and in the development of cervical cancer. Thus, epidemiological and biological understanding of the natural history of HPV infection is critical to guide the implementation of strategies for prevention and control of cervical cancer.

## Figures and Tables

**Table 1:** Evolution of HPV infection related to virus oncogenic risk and type of infection.

	Total *N*	Clearance *n* (%)	Persistence *n* (%)	*P*
Infection				
Single	80	34 (42.5)	46 (57.5)	0.210
Multiple	9	2 (22.2)	7 (77.8)	
Oncogenic risk				
LR-HPV	17	5 (29.4)	12 (70.6)	0.082
IR-HPV	17	10 (58.8)	7 (41.2)	
HR-HPV	55	21 (38.2)	34 (61.8)	

**Table 2:** Persistence or clearance of specific high-risk (HR) HPV infection after follow-up.

HPV types	Clearance *n* (%)	Persistence *n* (%)	Total *N*
16	12 (35.3)	22 (64.7)	34
18	3 (50)	3 (50)	6
31	2 (100)	0 (0)	2
33	0 (0)	2 (100)	2
45	1 (100)	0 (0)	1
53	1 (50)	1 (50)	2
58	1 (100)	0 (0)	1
59	0 (0)	1 (100)	1
66	0 (0)	1 (100)	1
67	1 (50)	1 (50)	2
69	0 (0)	1 (100)	1
83	0 (0)	2 (100)	2
HR-HPV	21 (38.2)	34 (61.8)	55

**Table 3:** Persistence or clearance of specific low-risk (LR) HPV infection after follow-up.

HPV types	Clearance *n* (%)	Persistence *n* (%)	Total *N*
6	4 (66.7)	2 (33.3)	6
11	0 (0)	2 (100)	2
61	1 (14.3)	6 (85.7)	7
72	0 (0)	1 (100)	1
81	0 (0)	1 (100)	1
LR-HPV	5 (29.4)	12 (70.6)	17

**Table 4:** Evolution of HPV infection related to sociodemographic and behavioral characteristics.

	Total *N*	Clearance *n* (%)	Persistence *n* (%)	*P*
Age groups (years)				
>30	28	11 (39.3)	17 (60.7)	0.657
30 to 39	20	7 (35.0)	13 (65.0)	
40 to 49	25	11 (44.0)	14 (56.0)	
>50	16	7 (40.4)	9 (56.3)	
Residence				
Rural	21	10 (47.6)	11 (52.4)	0.303
Urban	68	26 (38.2)	42 (61.8)	
Age at first intercourse (years)				
≤15	18	8 (44.4)	10 (56.6)	0.449
>15	71	28 (39.4)	43 (60.6)	
Number of lifetime sexual partners				
1	46	18 (39.1)	28 (60.9)	0.051
2 to 4	28	8 (28.6)	20 (71.4)	
5 or more	15	10 (66.7)	5 (33.3)	
Oral contraceptive use				
No	47	19 (40.4)	28 (59.6)	0.583
Yes	42	17 (40.5)	25 (59.5)	
Parity				
0	19	8 (42.1)	11 (57.9)	0.509
1 to 5	59	25 (42.4)	34 (57.6)	
6 or more	11	3 (27.3)	8 (72.7)	
Bracken fern consumption				
No	28	12 (42.9)	16 (57.1)	0.466
Yes	61	24 (39.3)	37 (60.7)	
Smoking				
No	53	19 (35.8)	34 (64.2)	0.197
Yes	36	17 (47.2)	19 (52.8)	
Alcohol consumption				
No	34	13 (38.2)	21 (61.8)	0.457
Yes	55	23 (41.8)	32 (58.2)	
